# Multiple primary hydatid cysts in the left thigh

**DOI:** 10.1590/0037-8682-0345-2022

**Published:** 2022-10-24

**Authors:** Kutsi Tuncer, Mehmet Demir, Yener Aydin

**Affiliations:** 1 Altıntas University, Medical Faculty, Department of Orthopedics and Traumatology, Istanbul, Turkey.; 2 Erzurum Regional Education and Research Hospital, Department of Orthopedics and Traumatology, Erzurum, Turkey.; 3 Ataturk University, Medical Faculty, Department of Thoracic Surgery, Erzurum, Turkey.

**FIGURE 1:**
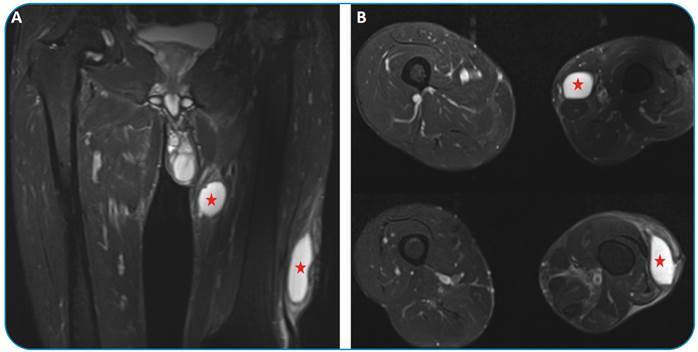
Coronal **(A)** and axial **(B)** fat-suppressed proton density MR images show lesions compatible with hydatid cysts 55 × 25 × 75 mm in the left knee superolateral part and 30 × 25 × 45 mm at the adductor muscle group plans (asterisks).

A 51-year-old male patient presented with erythema and swelling in the lateral part of the left knee. He had a history of working with livestock. Magnetic resonance imaging (MRI) showed lesions compatible with hydatid cysts in the superolateral part of the left knee and at the adductor muscle group plans (Figure 1). There was no hydatid cyst in the body except in the left thigh. Hydatid cysts were resected, and albendazole treatment (15 mg/kg/day) was administered for 3 months postoperatively.

Hydatid cysts, a zoonotic infection caused by *Echinococcus granulosus* larvae, is a zoonotic disease of major clinical importance^1^. Hydatid cysts are rare in muscle and subcutaneous areas^2^. MRI is considered the best technique for revealing daughter cysts and provides the most appropriate data for planning the surgical approach^3^. In painless, growing masses, a diagnosis of hydatid cysts should be kept in mind, especially in endemic regions. If possible, total surgical excision should be performed in such patients.
